# Propionate Attenuates Growth of Oral Streptococci through Enhancing Methionine Biosynthesis

**DOI:** 10.4014/jmb.2205.05037

**Published:** 2022-09-01

**Authors:** Taehwan Park, Jintaek Im, A Reum Kim, Dongwook Lee, Sungho Jeong, Cheol-Heui Yun, Seung Hyun Han

**Affiliations:** 1 Department of Oral Microbiology and Immunology, and Dental Research Institute, School of Dentistry, Seoul National University, Seoul 08826, Republic of Korea; 2 Department of Agricultural Biotechnology, and Research Institute of Agriculture and Life Sciences, Seoul National University, Seoul 08826, Republic of Korea; 3 Institute of Green Bio Science and Technology, Seoul National University, Pyeongchang 25354, Republic of Korea

**Keywords:** Short-chain fatty acids, propionate, oral streptococci, bacterial growth, methionine

## Abstract

Oral streptococci are considered as an opportunistic pathogen associated with initiation and progression of various oral diseases. However, since the currently-available treatments often accompany adverse effects, alternative strategy is demanded to control streptococci. In the current study, we investigated whether short-chain fatty acids (SCFAs), including sodium acetate (NaA), sodium propionate (NaP), and sodium butyrate (NaB), can inhibit the growth of oral streptococci. Among the tested SCFAs, NaP most potently inhibited the growth of laboratory and clinically isolated strains of *Streptococcus gordonii* under anaerobic culture conditions. However, the growth inhibitory effect of NaP on six different species of other oral streptococci was different depending on their culture conditions. Metabolic changes such as alteration of methionine biosynthesis can affect bacterial growth. Indeed, NaP enhanced intracellular methionine levels of oral streptococci as well as the mRNA expression level of methionine biosynthesis-related genes. Collectively, these results suggest that NaP has an inhibitory effect on the growth of oral streptococci, which might be due to alteration of methionine biosynthesis. Thus, NaP can be used an effective bacteriostatic agent for the prevention of oral infectious diseases caused by oral streptococci.

## Introduction

Dental caries and periodontitis are the most common oral diseases globally, putting tremendous burdens on public health sector. It has been reported that at least 20% of dental caries are left untreated, and periodontitis cases soared up to 743 million worldwide [1]. Moreover, oral diseases could lead to chronic systemic diseases such as diabetes mellitus, cardiovascular disease, cerebrovascular disease, and dementia [2]. Expenses spent for treating oral diseases were estimated to be 442 billion US dollars per year globally [3]. Much of the blame for those relentless oral diseases has been attributed by oral microorganisms such as streptococcus species being the main culprits [4, 5].

Streptococci, Gram-positive commensal bacteria, are frequently found in the human body, such as oral cavity, skin, and upper respiratory tract [6]. However, streptococci can also act as opportunistic pathogens involved in initiation and progression of various human infectious diseases, including pneumonia, sepsis, and skin infections [7]. Among various streptococci, *Streptococcus gordonii*, *Streptococcus sanguinis*, *Streptococcus oralis*, *Streptococcus salivarius*, *Streptococcus mitis*, *Streptococcus sobrinus*, and *Streptococcus mutans* are considered as major oral streptococci commonly found in patients with oral diseases such as periodontitis, dental caries, and gingivitis, implicating their pathological roles in developing the diseases [6-8]. Currently, various methods to remove oral streptococci are clinically employed. For instance, physical removal by tooth brushing or scaling is commonly used to physically detach oral streptococci. On the other hand, chemical approaches, such as antibiotics, calcium hydroxide, and chlorhexidine, are used in dental practice and root canal treatment for eliminating oral pathogens [9, 10]. However, the aforementioned methods often accompany various adverse effects. For instance, physical removal often causes gingival recession [11] while chemical treatment can weaken dentine strength, generate antibiotic-resistant bacteria, cause dry mouth, and induce taste change [12-14]. Hence, alternative strategies for controlling oral streptococci with marginal adverse effects are in demand to efficiently prevent and/or treat oral diseases.

Short-chain fatty acids (SCFAs) are produced mainly as bacterial metabolites, which are the by-products of dietary fibers and carbohydrates fermentation [15]. The representative SCFAs, acetate, propionate, and butyrate, account for more than 95% of all SCFAs and exist in a molar ratio of roughly 60:20:20, respectively, in human colon [16]. In oral cavity of human, 6.3-16.2 mM acetate, 1.2-3.1 mM propionate, and 0.0-0.4 mM butyrate are present [17]. Although many commensal bacteria can produce SCFAs in diverse pathways, the type of SCFAs produced is dependent on the bacterial species. For example, acetate and propionate are mainly produced by Bacteroidetes while butyrate is produced by Firmicutes [18]. Associated with their biological roles, SCFAs are mainly served as energy sources for certain bacteria. For example, butyrate produced by *Prevotella* and *Porphyromonas* species in dental plaque is used as an energy source for *Treponema* species [19]. Moreover, SCFAs are involved in regulation of host immune responses. For instance, butyrate enhanced oral cholera vaccine-induced CCL20 chemokine production in human intestinal epithelial cells [20]. In addition, butyrate attenuated nitric oxide production of macrophages induced by *Staphylococcus aureus* lipoproteins [21].

Furthermore, anti-microbial effect of SCFAs against various pathogenic bacteria has been reported. For instance, acetate inhibits the growth of *Escherichia coli* via altering acetate metabolism [22], while butyrate limits the growth of *Helicobacter pylori* by damaging its cell envelope [23]. Moreover, we previously reported that propionate attenuates *S. aureus* skin infection by inhibiting its growth [24] and has a bacteriostatic effect on *Enterococcus faecalis*, which is considered as a pathogen related to pathogenicity of apical periodontitis [25]. However, the effect of SCFAs on the growth of oral streptococci has not been extensively studied. Therefore, in this study, we investigated the effects of SCFAs on the growth of oral streptococci under various culture conditions and characterized their underlying mechanisms.

## Materials and Methods

### Reagent and Chemicals

SCFAs, including sodium acetate (NaA), sodium propionate (NaP), and sodium butyrate (NaB), and L-methionine were purchased from Sigma-Aldrich Inc. (USA). SCFAs and L-methionine were dissolved in distilled water, and sterilized by filtration with a syringe filter (0.2 μm pore size; Corning, USA). Todd-Hewitt (TH), yeast extract, and Bacto agar were purchased from BD Biosciences (USA).

### Bacteria Strains and Culture Conditions

A total of 12 strains of oral streptococci, including 6 strains of *S. gordonii*, and a strain for each of *S. sanguinis*, *S. oralis*, *S. mitis*, *S. salivarius*, *S. sorbrinus*, and *S. mutans*, were used to determine the effect of SCFAs on their growth. As shown in Table 1, laboratory strain of *S. gordonii* CH1 was kindly provided by Prof. Paul Sullam at University of California at San Franscisco (USA), and five clinically isolated strains of *S. gordonii*, recovered from dental plaque or oral cavity, were obtained from the Korean Collection for Oral Microbiology (Korea). Each laboratory strain of other oral streptococci was provided by Korean Collection for Type Cultures (Korea), Korea Atomic Energy Research Institute (Korea), or Prof. Bong-Kyu Choi at Seoul National University (Korea). All oral streptococci were grown in THY broth (TH broth containing 0.5% yeast extract) at 37°C under aerobic or anaerobic culture conditions using an anaerobic workstation (Whitley DG250, Don Whitley Scientific, UK).

### Bacterial Growth

A single colony of each oral streptococcal species grown in THY agar plate (THY broth containing 1.5% Bacto agar) was cultured in THY broth at 37°C overnight. Then, one percent of the culture was then inoculated to fresh THY broth in the presence or absence of various concentrations (0, 12.5, 25, 50, or 100 mM) of SCFAs in 15 ml conical tubes (SPL life science. Korea). The oral streptococci were cultured at 37°C with or without shaking (180 rpm/min) under aerobic or anaerobic culture conditions. The bacterial growth was estimated by measuring optical density at 600 nm at 0, 1, 3, 6, 9, 12, and 24 h using a microplate reader (SPARK, Tecan, Swiss).

### Minimum Inhibitory Concentration/Minimum Bactericidal Concentration (MIC/MBC) Test

The MIC/MBC test was performed as previously described [24] with minor modification. Briefly, one percent of the overnight cultured *S. gordonii* CH1 was inoculated to THY broth in the presence or absence of serially diluted NaP. The bacteria were cultured at 37°C for 24 h with or without shaking under aerobic or anaerobic culture conditions. After culturing the bacteria, optical density at 600 nm was measured using a microplate reader (SPARK, Tecan). MIC was defined as the minimum concentration of NaP needed for non-visible growth of *S. gordonii* at 24 h. To estimate MBC of NaP, the bacteria cultures showing low or non-bacterial growth from MIC test were dropped on NaP-free THY agar plate and incubated at 37°C for 24 h under anaerobic or aerobic condition. After the incubation, colony formation on the agar plate was examined.

### Measurement of pH

One percent of the overnight cultured *S. gordonii* CH1 was inoculated to THY broth in the presence or absence of NaP at 100 mM in 15 ml conical tubes. Bacteria were cultured at 37°C for various time periods (1, 3, 6, 9, 12, and 24 h) under anaerobic static culture conditions. After the incubation, the culture supernatants were dropped on reference pH strip (Whatman plc., UK), and pH changes were determined by colorimetric changes of the reference pH strip.

### Real-Time Polymerase Chain Reaction (PCR)

Real-time PCR was conducted as previously described [26] with minor modification. Briefly, *S. gordonii* CH1 was cultured in THY broth containing 100 mM NaP at 37°C for 24 h under anaerobic static culture conditions. The bacteria were harvested by centrifugation at 7,000 ×*g* for 10 min and suspended in nuclease-free water containing 10 mg/ml lysozyme (Sigma-Aldrich Inc.). After incubation at 37°C for 1 h, total RNA was isolated from bacterial lysate using TRIzol (Invitrogen, USA) according to the manufacturer’s instruction. After purifying total RNA using RNeasy mini kit (Qiagen), two micrograms of total RNA were subjected to complementary DNA synthesis using random hexamers and reverse transcriptase (Promega, USA). Then, the mRNA expressions of *metA*, *metE*, *SGO_1635*, *SGO_1636*, and 16S rRNA were determined by real-time PCR analysis using StepOnePlus real-time system (Applied Biosystems, USA) under the following reaction conditions: initial denaturation at 95ºC for 10 min, then amplification by 40 cycles of 95ºC for 15 s; 60ºC for 1 min. 16S rRNA was used as an internal control to normalize the target gene mRNA expression. Details of PCR primers used in the current study are listed in Table 2.

### Intracellular Methionine Level

Intracellular methionine levels were determined using a commercial methionine assay kit (Abcam, UK) according to the manufacturer’s instruction. Oral streptococci, including *S. gordonii* CH1, *S. sanguinis* KCTC 3284, *S. oralis* KCTC 13048, *S. mitis* SF-100, *S. salivarius* KCTC 3960, *S. sobrinus* 6715-7, and *S. mutans* KCTC 3065, were cultured in THY broth in the presence of various concentrations (0, 25, 50, and 100 mM) of NaP at 37°C for 24 h under anaerobic static culture condition. The bacteria were harvested by centrifugation at 7,000 ×*g* for 10 min and subsequently lysed by sonication on ice for 5 min. After collecting the bacterial lysates by centrifugation at 13,000 ×*g* for 8 min, 20 μl of bacterial lysate was mixed with 80 μl of reaction mixture in a 96-well plate. After the incubation at 37°C for 30 min, intracellular methionine levels were measured at 535 nm (Excitation)/587 nm (Emission) of fluorescence using a microplate reader (SPARK 10M, Tecan). The intracellular methionine level was presented as mM using a linear regression obtained from methionine standard from the kit.

### Statistical Analysis

All experiments were conducted at least three times, and the mean value ± standard deviation (SD) was determined from triplicate samples for each treatment group. Statistical significance was examined by Student’s *t*-test; asterisks (*) indicate experimental groups that are significantly different (*p* < 0.05) from the designated control group.

## Results

### SCFAs Suppress the Growth of *S. gordonii* under Anaerobic Culture Condition

**Fig. 1:**
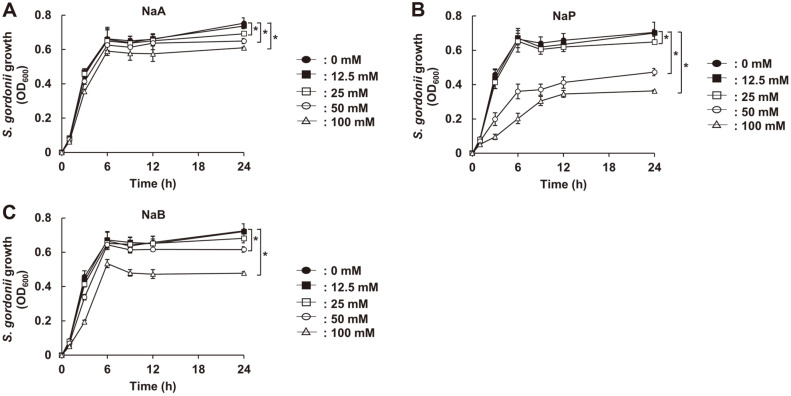
SCFAs suppress the growth of *S. gordonii* under anaerobic culture condition. *S. gordonii* CH1 was cultured in the presence or absence of various concentrations of (**A**) NaA, (**B**) NaP, or (**C**) NaB at 37°C for 24 h under anaerobic static culture condition. Optical density at 600 nm was then measured at 0, 1, 3, 6, 9, 12, and 24 h. Data shown are the mean values ± SD of triplicate samples and are representative of at least three similar independent experiments. *, *p* < 0.05 compared with the non-treatment group at 24 h.

**Fig. 2:**
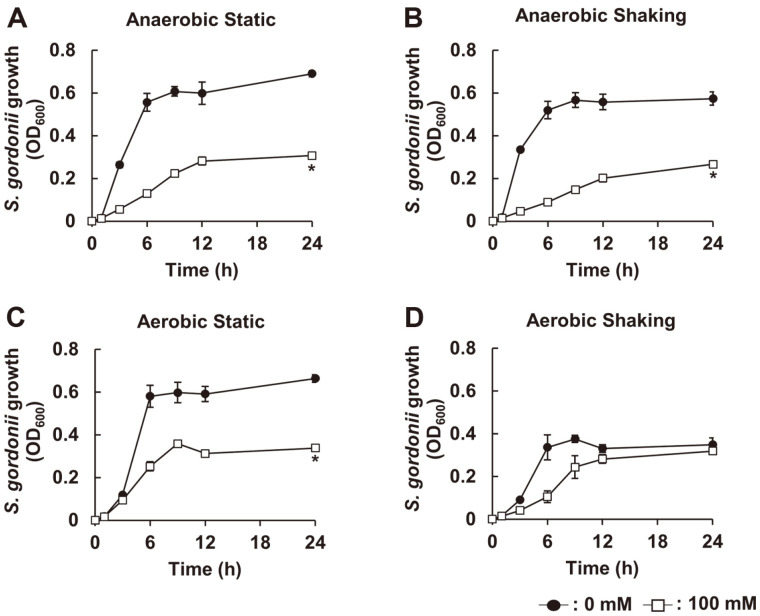
The growth inhibitory effect of NaP on *S. gordonii* is potent under anaerobic culture condition. *S. gordonii* CH1 was cultured in the absence or presence of NaP at 100 mM at 37°C for 24 h under (**A**) anaerobic static, (**B**) anaerobic shaking, (**C**) aerobic static, or (**D**) aerobic shaking culture conditions. Optical density at 600 nm was then measured at 0, 1, 3, 6, 9, 12, and 24 h. Data shown are the mean values ± SD of triplicate samples and are representative of at least three similar independent experiments. *, *p* < 0.05 compared with the non-treatment group at 24 h.

To examine the effect of SCFAs, including NaA, NaP, and NaB, on the growth of *S. gordonii*, we initially incubated *S. gordonii* CH1 in the presence or absence of various doses of SCFAs under anaerobic static condition and the growth was examined by measuring optical density at 600 nm at 0, 1, 3, 6, 12, and 24 h. As shown in Figs. 1A-1C, all tested SCFAs effectively inhibited *S. gordonii* growth in a dose-dependent manner. When comparing the growth inhibitory effects among SCFAs, NaP most potently inhibited the growth of *S. gordonii*, while NaA was the weakest. Furthermore, since the inhibitory property of NaP at 100 mM was most potently maintained up to 24 h, this concentration of NaP was used for further experiments. Next, we examined whether the growth inhibitory capacity of NaP on *S. gordonii* was affected by its culture condition. *S. gordonii* CH1 was cultured in the absence or presence of 100 mM NaP under four different culture conditions, including anaerobic static, anaerobic shaking, aerobic static, and aerobic shaking conditions, and their growth was examined. The inhibitory property of NaP under anaerobic culture condition was more potent than aerobic culture conditions (Figs. 2A-2D). Interestingly, the inhibitory property of NaP under aerobic static culture condition was almost abolished when cultured in aerobic shaking condition (Figs. 2C and 2D). Collectively, these results demonstrated that NaP, among the SCFAs, most potently suppressed the growth of *S. gordonii* under anaerobic culture condition.

### NaP Inhibits the Growth of *S. gordonii* through Bacteriostatic Effect

**Fig. 3:**
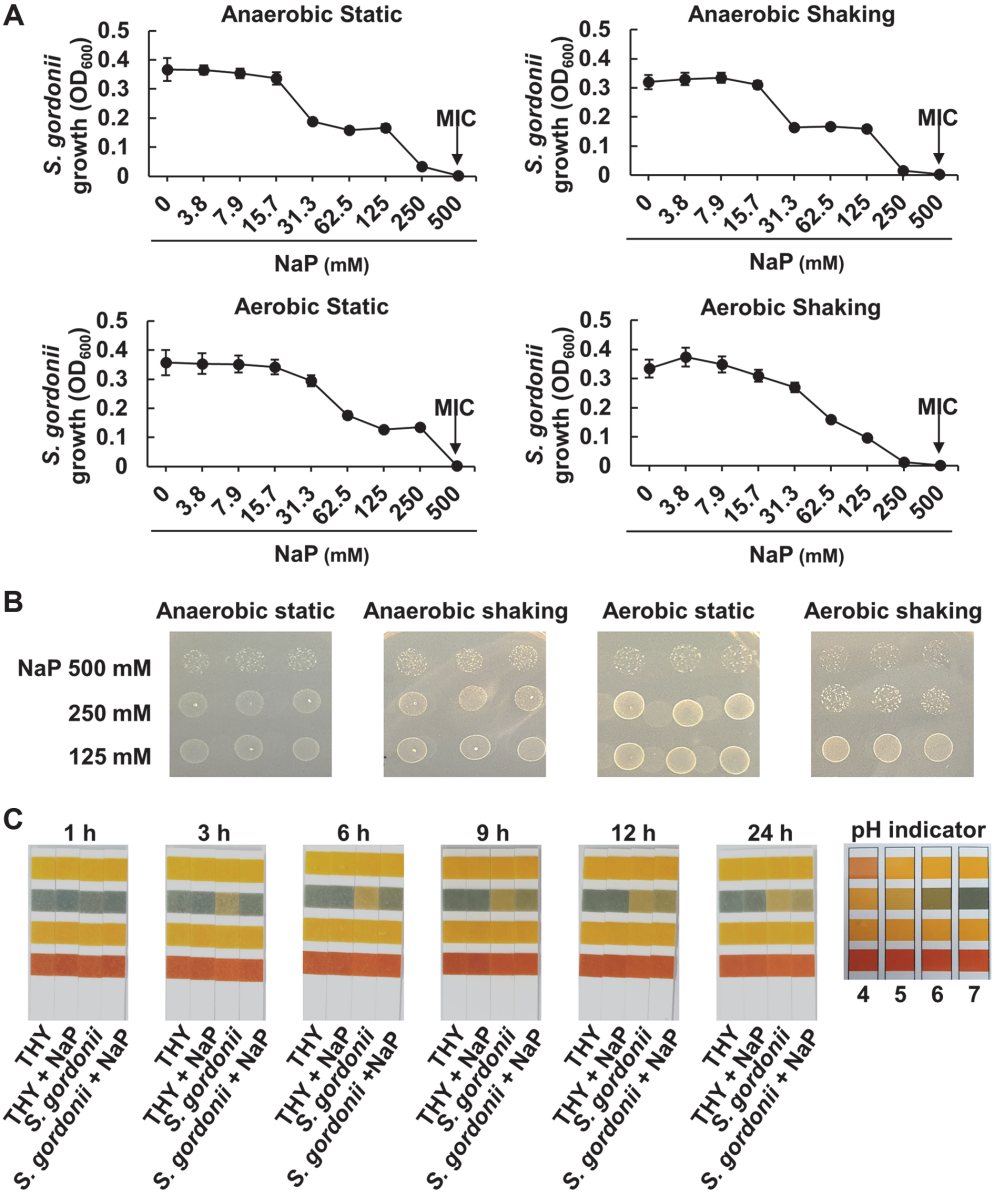
NaP inhibits the growth of *S. gordonii* through bacteriostatic effect. (**A**) *S. gordonii* CH1 was cultured in the presence of various concentrations of NaP at 37°C for 24 h under different culture conditions. After the incubation, optical density at 600 nm was measured and black arrow given in each figure indicates the MIC of NaP under each culture condition. Data shown are the mean values ± SD of triplicate samples and are representative of at least three similar independent experiments. (**B**) *S. gordonii* CH1 was incubated with 125, 250, or 500 mM of NaP at 37°C for 24 h under anaerobic static conditions. Then, cultured bacteria were dropped on NaP-free THY agar plate and incubated at 37°C for an additional 24 h. After the incubation, colony formation was enumerated. (**C**) *S. gordonii* CH1 was cultured in the presence or absence of 100 mM NaP at 37°C for various time periods under anaerobic static culture condition. The culture supernatants were then dropped on reference pH strip, and its colorimetric changes were examined.

To investigate whether the inhibitory capacity of NaP on *S. gordonii* growth is bacteriostatic or bactericidal, MIC and MBC of NaP were evaluated. For MIC determination for NaP, *S. gordonii* CH1 was cultured with various concentrations of NaP for 24 h under either anaerobic or aerobic culture condition. As shown in Fig. 3A, any bacterial growth of *S. gordonii* was not observed by 500 mM NaP treatment under all tested culture conditions, suggesting that this concentration is the MIC of NaP. Furthermore, MBC test was conducted by examining the colony formation of the bacteria cultures showing low or non-bacterial growth from MIC test under anaerobic and aerobic culture conditions. Since colony formation of *S. gordonii* was visible for all tested bacterial cultures even at 500 mM NaP, there was no MBC for NaP (Fig. 3B). We also tested if decreased bacterial growth was due to pH change induced by NaP. As shown in Fig. 3C, NaP supplementation (THY + NaP) did not affect pH in the culture medium. Moreover, the extracellular medium pH of *S. gordonii* incubated with NaP (*S. gordonii* + NaP) was maintained above pH 6 throughout the tested time periods. These results suggested that the growth inhibitory effects of NaP on *S. gordonii* is by its bacteriostatic rather than bactericidal effects.

### NaP Inhibits Growth of Clinically Isolated *S. gordonii* under Anaerobic Culture Condition

**Fig. 4:**
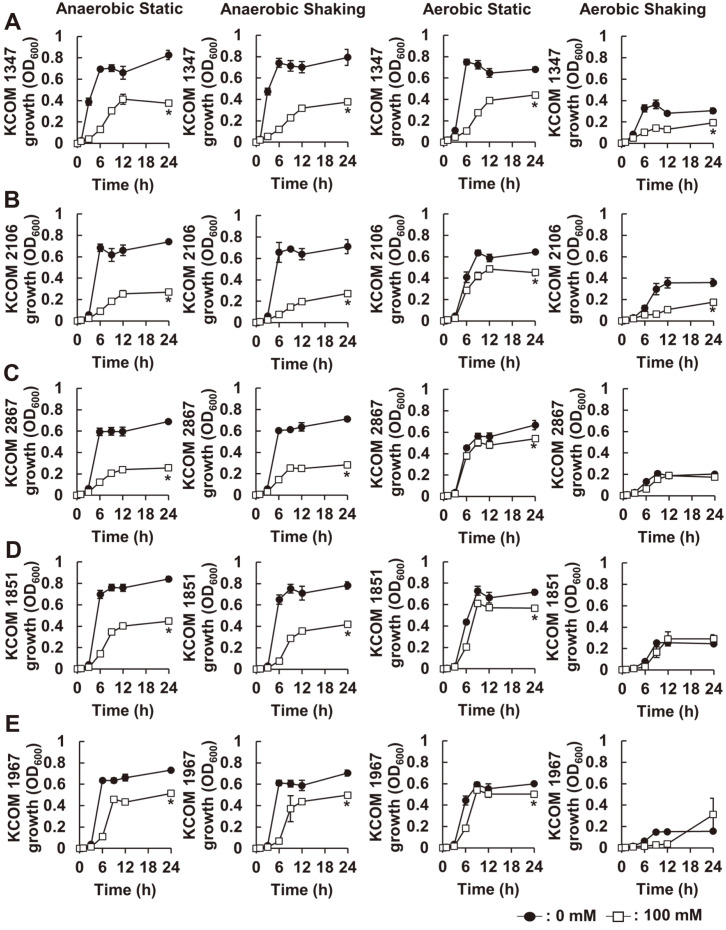
NaP inhibits growth of clinically isolated *S. gordonii* under anaerobic culture condition. Clinically isolated *S. gordonii* strains, including (**A**) KCOM 1347, (**B**) KCOM 2106, (**C**) KCOM 2867, (**D**) KCOM 1851, and (**E**) KCOM 1967 were cultured in the presence or absence of 100 mM NaP at 37°C for 24 h under various culture conditions. Optical density at 600 nm was measured at 0, 1, 3, 6, 9, 12, and 24 h. Data shown are the mean values ± SD of triplicate samples and are representative of at least three similar independent experiments. *, *p* < 0.05 compared with the non-treatment group at 24 h.

We next examined the growth inhibitory effects of NaP on five clinically isolated strains of *S. gordonii* from dental plaque or oral cavity. The clinical isolates of *S. gordonii*, including KCOM 1347, KCOM 2106, KCOM 2867, KCOM 1851, and KCOM 1967, were incubated in the presence or absence of 100 mM NaP for 24 h under various culture conditions, and their growth was examined by measuring optical density. Similar to the results in Fig. 2, NaP most potently inhibited the growth of all tested clinical isolates under anaerobic culture condition (Figs. 4A-4D). However, the inhibitory property of NaP under anaerobic culture condition was dramatically abolished under aerobic culture conditions. Especially, *S. gordonii* KCOM 2867, KCOM 1851, and KCOM 1967 were more resistant to NaP treatment under aerobic culture conditions than anaerobic culture conditions. These results suggested that the growth of clinically isolated *S. gordonii* is also inhibited by NaP treatment under anaerobic culture conditions.

### The Inhibitory Effect of NaP on Growth of Oral Streptococci is dependent on Culture Condition

**Fig. 5:**
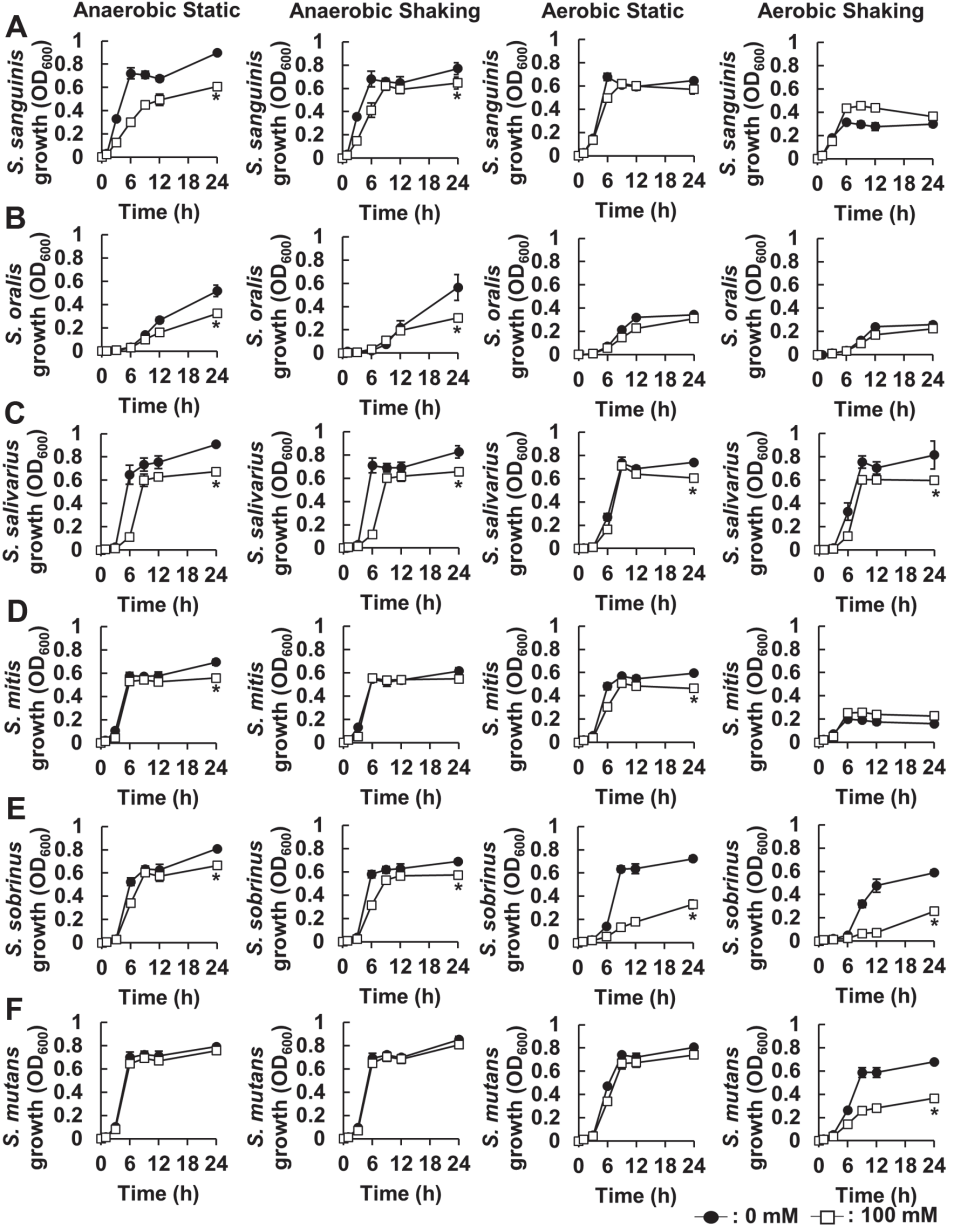
The inhibitory effect of NaP on growth of oral streptococci is dependent on culture condition. (**A**) *S. sanguinis* KCTC 3284, (**B**) *S. oralis* KCTC 13048, (**C**) *S. salivarius* KCTC 3960, (**D**) *S. mitis* SF-100, (**E**) *S. sobrinus* 6715-7, and (**F**) *S. mutans* KCTC 3065 were incubated in the presence or absence of 100 mM NaP at 37°C for 24 h under various culture conditions. Optical density at 600 nm was measured at 0, 1, 3, 6, 9, 12, and 24 h. Data shown are the mean values ± SD of triplicate samples and are representative of at least three similar independent experiments. *, *p* < 0.05 compared with the nontreatment group at 24 h.

Next, we evaluated the effects of NaP on growth of other oral streptococci, such as *S. sanguinis*, *S. oralis*, *S. salivarius*, *S. mitis*, *S. sobrinus*, and *S. mutans*. Laboratory strains of oral streptococci, including *S. sanguinis* KCTC 3284, *S. oralis* KCTC 13048, *S. salivarius* KCTC 3960, *S. mitis* SF-100, *S. sobrinus* 6715-7, and *S. mutans* KCTC 3065, were cultured in the absence or presence of 100 mM NaP for 24 h under both anaerobic and aerobic culture conditions, and their growth was measured. As shown in Figs. 5A-5F, the inhibitory effect of NaP on the growth of oral streptococci varied by culture conditions. The inhibitory effect of NaP on *S. sanguinis* and *S. oralis* was observed under anaerobic culture conditions while NaP barely affected their growth in aerobic culture conditions (Figs. 5A and 5B). In contrast, *S. sobrinus* and *S. mutans* growth were inhibited by NaP under aerobic conditions, but not anaerobic culture condition (Figs. 5E and 5F). In addition, the growth of *S. salivarius* was similarly suppressed by NaP under both anaerobic and aerobic culture conditions (Fig. 5C). Interestingly, *S. mitis* growth was rarely affected by NaP treatment under both anaerobic and aerobic culture conditions (Fig. 5D). Collectively, the results implied that the inhibitory effect of NaP on oral streptococci growth was generally dependent on the content of oxygen in the culture condition.

**Fig. 6:**
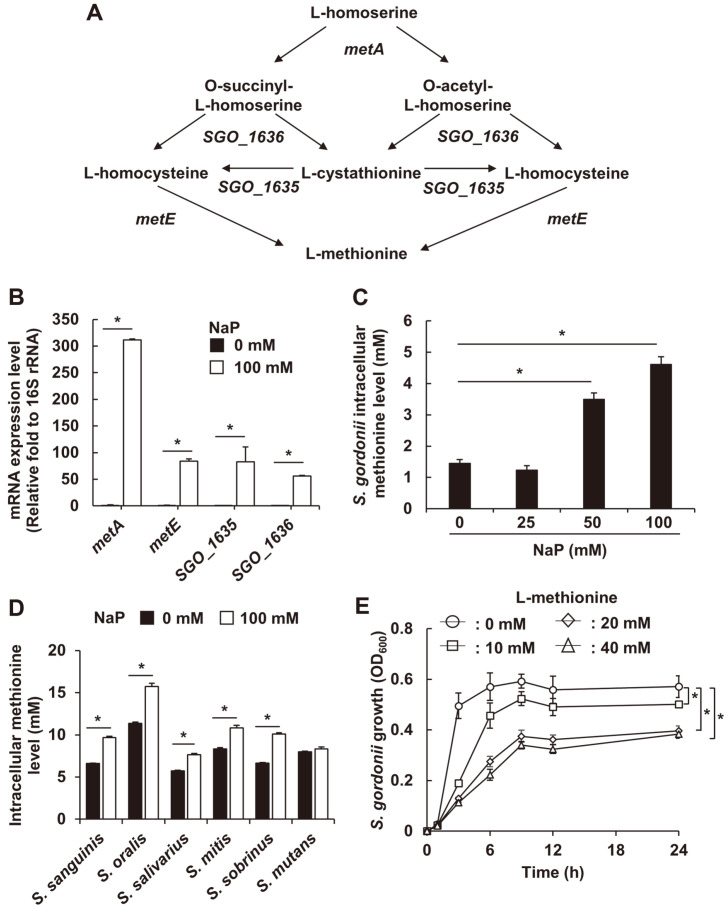
The growth inhibitory effect of NaP on *S. gordonii* is mediated by upregulated methionine synthesis. (**A**) The illustration of methionine biosynthesis pathway in *S. gordonii*. Gene names are indicated in italics. (**B**) Total RNA was isolated from *S. gordonii* CH1 cultured in the presence or absence of 100 mM NaP at 37°C for 24 h under anaerobic static culture condition. After the cDNA synthesis, real-time PCR analysis was conducted to examine mRNA expression levels of *metA*, *metE*, *SGO_1636*, and *SGO_1635* as described in Materials and Methods section. Data shown are the mean values ± SD of triplicate samples presented in the fold change of each target gene/16S rRNA ratio against non-treatment group set as 1-fold. *, *p* < 0.05 compared with the non-treatment group. (**C**) *S. gordonii* CH1 and (**D**) other oral streptococci including *S. sanguinis* KCTC 3284, *S. oralis* KCTC 13048, *S. salivarius* KCTC 3960, *S. mitis* SF-100, *S. sobrinus* 6715-7, and *S. mutans* KCTC 3065 were cultured in the presence of various concentrations of NaP at 37°C for 24 h under anaerobic static culture condition. Intracellular methionine levels using bacterial lysates were determined using a commercial methionine assay kit as described in Materials and Methods section. Data shown are the mean values ± SD of triplicate samples. *, *p* < 0.05 compared with the non-treatment group in each bacterial species. (**E**) *S. gordonii* CH1 was incubated in the presence or absence of various concentrations of Lmethionine at 37°C for 24 h under anaerobic static culture condition. Optical density at 600 nm was measured at 0, 1, 3, 6, 9, 12, and 24 h. Data shown are the mean values ± SD of triplicate samples and are representative of at least three similar independent experiments. *, *p* < 0.05 compared with the nontreatment group at 24 h.

The Growth Inhibitory Effect of NaP on Oral Streptococci is Mediated by Upregulated Methionine Synthesis Accumulated reports have suggested that propionate-mediated bacterial growth suppression might derive from altered metabolisms. In fact, propionate inhibited the growth of *Rhodopseudomonas sphaeroides* via suppression of its glycolysis metabolism [27]. Moreover, propionate and acetate inhibited E. coli growth through alteration of methionine synthesis metabolism [28]. Therefore, we examined whether NaP affects methionine synthesis of *S. gordonii*, leading to suppression of bacterial growth. Based on KEGG (*i.e.*, Kyoto Encyclopedia of Genes and Genomes) pathway database, the predicted methionine biosynthesis pathway in *S. gordonii* and the pathway-related genes are shown in Fig. 6A. Thus, the mRNA expression levels of methionine synthesis related genes, including *metA*, *SGO_1636*, *SGO_1635*, and *metE* were determined by real-time PCR analysis. As shown in Fig. 6B, gene expression levels of the methionine synthesis related genes in *S. gordonii* CH1 were significantly upregulated by 100 mM NaP treatment under anaerobic static culture condition. Furthermore, the intracellular methionine levels of *S. gordonii* CH1 were enhanced by NaP treatment under anaerobic static culture condition in a dose-dependent manner (Fig. 6C). On the other hand, intracellular methionine levels of *S. sanguinis* KCTC 3284, *S. oralis* KCTC 13048, *S. salivarius* KCTC 3960, *S. mitis* SF-100, and *S. sobrinus* 6715-7 were significantly increased by NaP treatment under anaerobic static culture condition. However, intracellular methionine level of *S. mutans* KCTC 3065 was not affected by NaP treatment (Fig. 6D). Collectively, these results showed that approximately 5-15 mM of intracellular methionine was generated by 100 mM NaP treatment in oral streptococci (Figs. 6C and 6D). To investigate the effect of methionine on its growth, *S. gordonii* CH1 was cultured in the presence or absence of various concentrations of L-methionine under anaerobic static culture condition. As shown in Fig. 6E, exogenous L-methionine treatment suppressed the growth of *S. gordonii* in a dose-dependent manner. To determine whether NaP suppresses its growth by impairing the glycolysis metabolism, we examined effect of exogenous pyruvate, which is the final metabolic product of glycolysis, on *S. gordonii* growth in the presence of NaP. However, exogenous pyruvate did not rescue NaP-induced growth inhibition of *S. gordonii*, suggesting that glycolysis metabolism is not relevant to NaP-induced growth inhibition (data not shown). Collectively, these results demonstrated that inhibited growth of oral streptococci by NaP is mediated via the increased intracellular methionine level.

## Discussion

Oral streptococci are closely related to pathogenicity of various oral diseases such as dental caries and periodontitis. However, the treatment in clinical practice often accompanies various adverse effects, such as soft tissue damage and reduced tooth strength. Therefore, alternative strategies for controlling oral streptococci with marginal adverse effects are needed. In the current study, we demonstrated that NaP, among the tested SCFAs, most potently inhibited the growth of both clinical isolates and laboratory strains of *S. gordonii* under anaerobic culture condition. Although the growth inhibitory property of NaP on other oral streptococci differed depending on their culture conditions, growth of most oral streptococci was suppressed by NaP treatment under anaerobic culture condition. Furthermore, mechanism studies suggest that the growth inhibitory effect of NaP is mediated through promoting methionine biosynthesis of oral streptococci.

Although all tested SCFAs showed the growth inhibitory effects against *S. gordonii*, their inhibitory effects differed depending on the type of SCFAs. In fact, NaP potently inhibited the growth of *S. gordonii*, while NaA showed relatively weaker growth inhibitory effect. These different growth inhibitory effects among SCFAs seem to be bacterial species-specific. In accordance with our observation, NaP more effectively inhibited the growth of *Pseudomonas aeruginosa* than equal concentration of NaA [29]. In contrast, NaA more potently suppressed the growth of E. coli than NaP [28]. These different growth inhibitory effects may result from differences in their intracellular diffusion rate. In fact, NaA, which has high intracellular diffusion rate, has more potent bacteriostatic effect than NaP having relatively lower intracellular diffusion rate in E. coli [30]. However, since it has been reported that NaP and NaA have similar intracellular diffusion rates into Gram-positive bacteria, such as *S. gordonii* [31], the different growth inhibitory effects of SCFAs on *S. gordonii* may not be caused by differences in their intracellular diffusion rate. On the other hand, the different growth inhibitory effects among SCFAs may be explained by metabolic characteristics of *S. gordonii*. Since oral streptococci possess acetate kinase which converts acetate into acetyl phosphate [32], *S. gordonii* can eliminate and further utilize the infiltrated acetate as a carbon source for their growth [33]. However, oral streptococci do not possess any metabolic system to remove and utilize the infiltrated butyrate and propionate, such as butyrate kinase and propionyl-CoA, which are found in *Clostridium acetobutylicum* and *Mycobacterium tuberculosis*, respectively [32, 34, 35]. Therefore, the presence of acetate converting enzyme of *S. gordonii* may provide enhanced resistance against NaA compared to other SCFAs, leading to lower growth inhibitory effect.

Although NaP potently inhibited the growth of most oral streptococci, including *S. gordonii*, under anaerobic culture condition, such inhibitory effect was weak in aerobic culture condition compared with anaerobic culture condition. Similar to our results, the growth inhibitory effect of NaP on *Listeria monocytogenes* under anaerobic culture condition was reduced under aerobic culture condition [36]. Based on previous reports, this phenomenon can be explained by the alteration of acid tolerance in streptococci depending on culture conditions. According to previous study, streptococci, such as *S. gordonii*, can utilize arginine deiminase system (ADS) as their acid tolerance mechanism. During arginine metabolism, ADS converts arginine into ammonia and citrulline, and the produced ammonia during the reaction then neutralizes acids such as propionate [37]. Furthermore, it has been reported that ADS activity is enhanced under aerobic condition by upregulation of its key regulator activity, FNR-like regulator (FlpS), which is more activated in the presence of oxygen [38]. Therefore, increased acid tolerance of oral streptococci by up-regulated ADS under aerobic culture condition may provide more increased resistance to NaP, leading to reduced bacteriostatic effect of NaP under aerobic conditions.

In the current study, we found that the growth inhibitory capacity of NaP on oral streptococci is positively correlated with their methionine synthesis induced by NaP. Although the growth inhibitory effect of methionine was previously reported in various microbes such as *Thiobacillus neapolitanus* and *Saccharomyces cerevisiae* [39, 40], the underlying mechanism on how enhanced methionine synthesis inhibited their growth is still unclear. However, accumulated studies may suggest a few possibilities. First, increased methionine levels can cause the accumulation of cytotoxic metabolic intermediator, homocysteine, leading to inhibition of oral streptococci growth. Although homocysteine is metabolized by methionyl tRNA synthetase under normal conditions, increased intracellular methionine levels can block this reaction by competing with homocysteine for binding to the enzyme [41]. Since homocysteine is also known as a metabolic intermediator in methionine biosynthesis metabolism of streptococci, NaP-induced methionine may accumulate cytotoxic homocysteine. Second, since methionine can directly down-regulate expression of various growth-related genes, such as the transcriptional regulator, GlnR, ABC transporter, pyruvate oxidase, and acetyl-CoA carboxylase [42], NaP-induced methionine synthesis may down-regulate those gene expressions leading to suppression of oral streptococci growth. Thus, further studies are needed to characterize the underlying mechanisms on how NaP-induced methionine inhibits the growth of oral streptococci based on the aforementioned hypotheses.

Oral streptococci are major bacteria frequently found in patients with various oral diseases such as periodontitis, dental caries, and gingivitis, suggesting their pathogenic roles in initiation and progression of such oral diseases [6-8]. Moreover, oral streptococci act as early colonizers providing habitats for other oral pathogenic bacteria, such as *Fusobacterium nucleatum*, *Porphyromonas gingivalis*, and *Aggregatibacter actinomycetemcomitans*, thereby forming multispecies biofilm leading to enhanced resistance against antibiotics and disinfectants [43]. Needless to say that controlling oral streptococci is important for maintenance of oral health. In this study, we demonstrated that NaP, among the tested SCFAs, has the most potent bacteriostatic effect on oral streptococci, including clinically isolated *S. gordonii*. Since propionate is known to exist in the human body, it is biocompatible and safe without extreme adverse effects than other conventional treatments. Therefore, our findings provide evidence that NaP could be applied as a useful bacteriostatic agent against oral streptococci for the prevention of oral diseases.

## Figures and Tables

**Table 1:** Oral streptococci strains used in the present study.

Bacteria species and strain	Source^a^
*Streptococcus gordonii*	
CH1	UCSF, laboratory strain
KCOM 1347	KCOM, clinical isolate (oral cavity)
KCOM 1851	KCOM, clinical isolate (oral cavity)
KCOM 1967	KCOM, clinical isolate (subgingival dental plaque)
KCOM 2106	KCOM, clinical isolate (subgingival dental plaque)
KCOM 2867	KCOM, clinical isolate (subgingival dental plaque)
*Streptococcus sanguinis* KCTC 3284	KCTC, laboratory strain
*Streptococcus oralis* KCTC 13048	KCTC, laboratory strain
*Streptococcus mitis* SF-100	KAERI, laboratory strain
*Streptococcus salivarius* KCTC 3960	KCTC, laboratory strain
*Streptococcus sobrinus* 6715-7	SNU, laboratory strain
*Streptococcus mutans* KCTC 3065	KCTC, laboratory strain

^a^UCSF, University of California at San Francisco; KCOM, Korean Collection for Oral Microbiology; KCTC, Korean Collection for Type Cultures; KAERI, Korea Atomic Energy Research Institute; SNU, Seoul National University.

**Table 2:** Sequences of primers used for real-time PCR analysis.

Target gene	Primer orientation	Primer sequence (5’-3’)	Product size (bp)
*metE*	Forward	GCGAAAAAGGAGATGTCCGC	148
	Reverse	CCTAGCTCCTTAGCTTCGGC	
*metA*	Forward	GCGTTGACAAGCACCAGATG	94
	Reverse	TCATCGTCAAAGCCACGGAA	
*SGO_1635*	Forward	CCCACCTTTTGCTCGCTCTA	77
	Reverse	AAAGGCCGTCTTGCTCTACC	
*SGO_1636*	Forward	CTGCACTCTGCGACCAAGTA	71
	Reverse	GCGTCATTGGTCATAACGGC	
16S rRNA	Forward	AAGCAACGCGAAGAACCTTA	198
	Reverse	GTCTCGCTAGAGTGCCCAAC	
